# Long-term outcome of neoadjuvant systemic therapy for locally advanced breast cancer in routine clinical practice

**DOI:** 10.1007/s00432-012-1325-9

**Published:** 2012-10-10

**Authors:** Domenico Angelucci, Nicola Tinari, Antonino Grassadonia, Ettore Cianchetti, Giampiero Ausili-Cefaro, Laura Iezzi, Marinella Zilli, Simona Grossi, Lucia Anna Ursini, Maria Teresa Scognamiglio, Graziella Castrilli, Michele De Tursi, Paolo Noccioli, Pasquale Cioffi, Stefano Iacobelli, Clara Natoli

**Affiliations:** 1 Division of Pathology, ‘SS. Annunziata’ Hospital, 66013 Chieti, Italy; 2 Division of Surgical Senology, Department of Experimental and Clinical Sciences, University ‘G. d’Annunzio’, 66013 Chieti, Italy; 3 Radiation Oncology Department, University ‘G. d’Annunzio’, 66013 Chieti, Italy; 4 Division of Surgical Senology, “G. Bernabeo” Hospital, 66026 Ortona, CH Italy; 5 Division of Radiation Oncology, ‘SS. Annunziata’ Hospital, 66013 Chieti, Italy; 6 Oncology Department, ‘SS. Annunziata’ Hospital, 66013 Chieti, Italy; 7 Division of Pathology, “G. Bernabeo” Hospital, 66026 Ortona, CH Italy; 8 Hospital Pharmacy, ‘SS. Annunziata’ Hospital, 66013 Chieti, Italy; 9 Department of Experimental and Clinical Sciences, University ‘G. d’Annunzio’, 66013 Chieti, Italy

**Keywords:** Breast cancer, Neoadjuvant chemotherapy, Retrospective, Pathological complete response, Prognostic factors

## Abstract

**Purpose:**

The aim of this study is to evaluate the long-term outcome of patients with locally advanced breast cancer treated with neoadjuvant systemic chemotherapy (NST) in routine clinical practice.

**Methods:**

Four hundred and nine patients were identified between January 1999 and December 2011. All patients received NST followed by surgery, adjuvant treatments and radiotherapy, as appropriate.

**Results:**

At Kaplan–Meier analysis, patients with surgical stage III disease were more likely to develop distant metastasis and die from breast cancer (*p* < 0.001). Luminal A and luminal B/HER2-negative patients had better prognosis; moreover, patients with hormone receptor (HR)-positive tumors had a significantly longer DRFS (*p* < 0.0049) and OS (*p* < 0.0001) compared with patients with HR-negative tumors as well as patients who underwent breast-conserving surgery (DRFS and OS: *p* < 0.001). In multivariate analysis, HR negativity (*p* < 0.001 for both DRFS and OS), mastectomy (DRFS: *p* = 0.009; OS: *p* = 0.05) and stage III disease (DRFS: *p* < 0.001; OS: *p* = 0.003) were associated with shorter DRFS and OS.

**Conclusions:**

HR negativity, mastectomy and pathological stage III disease are the variables independently associated with a worse outcome in our cohort of patients. These data are of high interest since they derive from a very heterogeneous group of patients, treated with different neoadjuvant/adjuvant regimens outside of clinical trials and with a long follow-up period.

## Background

Neoadjuvant systemic therapy (NST) has been used in locally advanced breast cancer in order to convert a previously unresectable cancer into an operable one (Hortobagyi et al. 1988; Danforth et al. 1990; Schwartz et al. 1994). More recently, it has been widely administered in primarily operable breast cancer to reduce tumor volume and allow conservative surgery (Fisher et al. 1997; van der Hage et al. 2001; Semiglazov et al. 2011). The downstaging of the primary tumor and the increase in breast conservation rates seems to be the only clinical benefit of NST, given that several studies failed to demonstrate an improvement of overall survival compared with postoperative adjuvant chemotherapy (Fisher et al. 1997, 1998; Bear et al. 2006; Mauri et al. 2005). Breast cancer is a heterogeneous disease that varies widely in response to standard therapies and outcomes (Rouzier et al. 2005). In this perspective, NST represents an opportunity to determine the intrinsic resistance/sensitiveness of breast cancer to chemotherapy. Moreover, the extent of residual disease in the breast and axillary surgical specimens after NST, classified according to the revised 2003 American Joint Committee on Cancer (AJCC) tumor-node-metastasis (TNM) staging system, has been reported to be associated with relapse and survival (Carey et al. 2005). Pathologic complete response (pCR) achieved after NST, when defined as non-invasive and non-in situ cancer in breast and nodes, is predictive of good prognosis and might be used as surrogate of survival (Kuerer et al. 1999; Kaufmann et al. 2006). However, patients with hormone receptors (HR)-positive tumors usually have low rates of pCR and maintain a good long-term outcome even in the presence of residual disease (no pCR) (Colleoni et al. 2009; Huober et al. 2010; Precht et al. 2010; Straver et al. 2010; Kim et al. 2010). In this group of patients, pCR fails to predict survival, emphasizing the importance of tumor biology rather than response to neoadjuvant chemotherapy as a prognostic marker in some subtypes of breast cancers (von Minckwitz et al. 2012).

The aim of this study is to evaluate the long-term outcome in a series of patients with locally advanced breast cancer consecutively treated with NST in our institution. All patients came from the routine clinical practice and were not included in clinical trials.

## Patients and methods

### Patient population

This is a single institution study. The charts of all patients with locally advanced breast cancer, performance status 0–2 (ECOG scale), consecutively treated with NST at the Medical Oncology Division, University of Chieti Hospital between January 1999 and December 2011, were reviewed for this retrospective study. Four hundred and nine patients were identified. In all cases, diagnosis of invasive breast cancer was established by tru-cut biopsy of the primary tumor. Patients with bilateral and inflammatory breast cancer were excluded.

All patients received preoperative chemotherapy and those with HR-positive tumor received adjuvant hormonal therapy for 5 years. Chemotherapy regimens administered included: CMF (fluorouracil, methotrexate and cyclophosphamide); single-agent epirubicin; EC (epirubicin and cyclophosphamide); FEC (fluorouracil, epirubicin and cyclophosphamide); E-CMF (single-agent epirubicin followed by CMF); single-agent taxanes; ET (epirubicin and taxol); EC-T (EC followed by docetaxel); EC-TAXEL (EC followed by docetaxel and capecitabine) and other combinations including platinum compounds, vinorelbine and pegylated doxorubicin. Ninety-nine patients received adjuvant tamoxifen, 125 postmenopausal patients received aromatase inhibitors (anastrozole or letrozole) and 71 patients tamoxifen followed by exemestane. Ninety-four patients treated after 2005 and carrying HER2-positive tumors received Trastuzumab simultaneously with neoadjuvant chemotherapy and/or postoperatively to complete 1 year of treatment.

Surgical procedures consisted of mastectomy or breast-conserving surgery (BCS). Sentinel node biopsy after NST was performed in 54 patients; axillary lymph node dissection was performed in 371 (90.1 %) patients, including 16 having positive sentinel nodes. Adjuvant breast radiotherapy was delivered to patients who underwent BCS as well as to patients who underwent mastectomy but had initial stage cT3, cN2 or cN3 disease.

### Pathological assessments

Estrogen (ER)/progesterone receptors (PR) and human epidermal growth factor type 2 receptor (HER2) were determined on pretreatment biopsy and on surgical specimens by immunohistochemistry. HR status was considered positive if ≥10 % of tumor cells stained for ER and/or PR. HER2 status was assessed by HercepTest (Dako Italia, Milan, Italy). Samples were scored as follows: score 0, membrane staining in ≤10 % of tumor cells; score 1+ , partial and/or faint membrane staining in >10 % of tumor cells; score 2+, weak to moderate, complete membrane staining in >10 % tumor cells and score 3+, strong, complete membrane staining in >10 % of tumor cells. FISH or CISH was carried out on all tumors with HercepTest 2+. Tumors with a score of 3+ by immunohistochemistry (IHC) or gene amplification by FISH were considered as HER2 positive. Immunohistochemical detection of Ki-67 was performed using the MIB-1 antibody (Dowsett et al. 2011). Nuclear grade was assessed according to the Nottingham grading system (Elston and Ellis 1991).

We could not exactly define breast cancer intrinsic subtypes with immunohistochemistry in all tumors (Goldhirsch et al. 2011), since Ki-67 assessment was not available in 172 (42.0 %) samples. So we classified tumors as follows: (1) luminal A and luminal B/HER2 negative; (2) luminal B/HER2 positive; (3) HER2 enriched; (4) triple negative (Houssami et al. 2012).

pCR was defined as non-invasive cancer within the breast (ypT0/is) and lymph node (ypN0), also classified as Stage 0 (Kuerer et al. 1999; Kaufmann et al. 2010). Pathological stages were categorized according to the American Joint Committee on Cancer Staging Manual, 7th ed. (Edge et al. 2010).

Locoregional recurrence (LRR) was defined as any chest wall recurrence in those who underwent mastectomy, any ipsilateral in-breast recurrence in those achieving breast conservation and any recurrence in the axillary, supraclavicular or internal mammary nodes.

### Data collection

Medical records for all patients were reviewed retrospectively and the cut off date for follow-up set on December 31, 2011. Clinical and pathological characteristics for each patient were entered on an anonymized database. Since patients’ enrollment began in 1999, complete information was not available for all 409 patients; thus, denominators may vary throughout the article. The follow-up contacts were carried out at 6-month intervals over the first 5 years, and at 12-month intervals thereafter.

### Study endpoints and statistics

The primary endpoint of this study was overall survival (OS), defined as the interval between the time of surgery and the date of death from any cause or censoring. Survivors were censored at the date of last contact. The secondary endpoints were rate of pCR and distant relapse—free survival (DRFS), defined as the time from breast surgery to the first occurrence of distant metastasis or intercurrent deaths without distant recurrence.

A two-sided level of significance of 0.05 was applied to all statistical tests. In univariate analysis, the relationships between patients/tumor characteristics and pCR were assessed by Pearson’s χ^2^ or Fisher’s exact test, as appropriate. A stepwise multivariate logistic regression was used to identify independent predictors of pCR among baseline patients/tumor characteristics. Survival curves were derived from Kaplan–Meier estimates and compared by log-rank test and hazard ratio (HR) (Massarweh et al. 2006). A multivariate Cox proportional hazard model was carried out to assess the relative influence of prognostic factors on survival (De Placido et al. 2003). All statistical analyses were performed using the SPSS Statistic software version 19 (IBM, Armonk, New York).

## Results

### Baseline patient and tumor characteristics

Clinical and pathological baseline characteristics of patients are shown in Table 1. The median age was 48.8 years (range 25–80), with 20 (4.9 %) patients being younger than 35 years and 15 (3.7 %) older than 70 years. Clinical tumor size was ≥3 cm in 175 (42.8 %) patients; 346 (84.6 %) patients had ductal carcinoma. Tumor grade was 1–2 in 289 (70.7 %) and grade 3 in 87 (21.2 %) patients. Ki-67 was available in 237 (58 %) cases and was >14 % in 135 (56.9 %). Tumors were classified in four molecular subtypes according to the tumor staining for ER/PR and HER2 status: 211 (51.9 %) were HER2 negative, luminal A or luminal B (ER and/or PR positive); 84 (20.6 %) were HER2 positive, luminal B (ER and/or PR positive); 53 (13.0 %) were HER2 enriched (ER and PR negative, HER2 positive); 59 (14.5 %) were triple negative (ER and PR negatives, HER2 negative). Most patients, 237 (58.0 %), received chemotherapy based on anthracycline and taxanes. Among 137 women with HER2-positive tumor, 43 (31.4 %), diagnosed before 2005, were not treated with Trastuzumab, 29 (21.2 %) received adjuvant Trastuzumab and 65 (47.4 %) received neoadjuvant and adjuvant Trastuzumab. A total of 300 (73.3 %) patients received more than four cycles of chemotherapy.

**Table 1:** Association of baseline factors and pCR in univariate analysis

	No. (%)	pCR no. (%)	*p* value
*Age*			
Median age 48.8 years (range 25–80 years)			
≤35 years	20 (4.9)	5 (25.0)	
>35 years	389 (95.1)	56 (14.4)	n.s.
*Clinical T*			
≤3 cm	213 (52.1)	30 (14.0)	
≥3 cm	175 (42.8)	30 (17.1)	
Unknown^a^	21 (5.1)	1 (4.8)	n.s.
*Histologic type*			
Ductal	346 (84.6)	51 (14.7)	
Lobular	57 (14.0)	10 (17.5)	
Others	6 (1.4)	0	n.s.
*Grade*			
1–2	289 (70.7)	25 (8.6)	
3	87 (21.2)	19 (21.8)	
Unknown^a^	33 (8.1)	17 (51.5)	0.001
*Ki*-*67*			
≤14 %	102 (43.1)	4 (3.9)	
>14 %	135 (56.9)	33 (22.2)	
Unknown^a^	172 (50.0)	24 (14.0)	0.000
*Molecular subtype*			
Luminal A & B/HER2 negative	211 (51.9)	12 (5.7)	
Luminal B/HER2 positive	84 (20.6)	14 (16.6)	
HER2 enriched	53 (13.0)	18 (33.9)	
Triple negative	59 (14.5)	17 (28.8)	
Unknown^a^	2 (0.04)		0.000
*Type of NST*			
Various	107 (26.1)	5 (4.5)*	
Anthracycline and taxane	237 (58.0)	30 (12.6)*	
Chemotherapy + Trastuzumab	65 (15.9)	26 (40.0)	0.000
*No. of chemotherapy cycles*			
≤4	109 (26.7)	7 (6.4)	
>4	300 (73.3)	54 (18.0)	0.004

* 10.2 % pCR in patients treated with chemotherapy only

^a^Unknown were not included in univariate analysis

### Relationship between baseline characteristics and pCR

In the univariate analysis, pCR was significantly associated with tumor grade, proliferative activity, molecular subtype, type of NST and number of chemotherapy cycles (Table 1), patients with the worst prognostic factors having the best pCR rates. In the multivariate analysis, only HR-negative tumors, independently from HER2 status (HER2 enriched: *p* = 0.043; triple negative: *p* = 0.002) and the use of neoadjuvant Trastuzumab (*p* = 0.035) were significantly associated with higher pCR rates (Table 2).

**Table 2:** Association of baseline factors and pCR in multivariate analysis

	Odds ratio	95 % CI	*p* value
*Age*			
>35 years		Reference	
≤35 years	2.081	0.426–10.175	n.s.
*Clinical T*			
≤3 cm		Reference	
≥3 cm	1.040	0.414–2.611	n.s.
*Histologic type*			
Other		Reference	
Ductal	2.965	0.327–6.424	n.s.
Lobular	5.995	0.213–10.785	n.s.
*Grade*			
1–2		Reference	
3	1.275	0.418–3.885	n.s.
*Ki*-*67*			
≤14 %		Reference	
>14 %	3.689	0.899–15.132	n.s.
*Molecular subtype*			
Luminal A and B/HER2 negative		Reference	
Luminal B/HER2 positive	1.564	0.252–9.716	n.s.
HER2 enriched	6.090	1.062–34.921	0.043
Triple negative	10.646	2.307–40.125	0.002
*Type of NST*			
Various	2.290	Reference	n.s.
Anthracycline and taxane	11.334	0.300–17.419	0.035
Chemotherapy + Trastuzumab		1.182–108.719	
*No. of chemotherapy cycles*			
≤4	2.158	Reference	n.s.
>4		0.308–15.143	

### Patients’ characteristics after NST

Patients’ characteristics after completion of NST are reported in Table 3. BCS was performed in 241 (58.9 %) patients and mastectomy in the remaining 168 (41.1 %). Absence of cancer in the breast (ypT0) was found in 75 (18.3 %) patients; absence of cancer in lymph nodes (ypN0) in 181 (44.2 %) patients and a total of 61 (14.9 %) patients had a pCR, that is, absence of invasive cancer both in breast and nodes. Most patients received adjuvant treatments: 125 (30.6 %) patients had only hormonal therapy, 54 (13.2 %) only chemotherapy, 105 (25.6 %) chemotherapy followed by hormonal therapy and 94 (23.0 %) received adjuvant Trastuzumab either alone (33 patients), with hormonal therapy (39 patients), with chemotherapy (11 patients) or with chemotherapy and hormonal therapy (11 patients). Adjuvant radiotherapy was delivered to 310 (75.8 %) patients, including 101 patients who underwent mastectomy.

**Table 3:** Clinical characteristics of patients after NST therapy

	No. (%)
*Type of surgery*	
BCS	241 (58.9)
Mastectomy	168 (41.1)
*Residual tumor size*	
ypT0	75 (18.3)
ypT1	180 (44.0)
ypT2	116 (28.3)
ypT3	38 (9.2)
*No. of metastatic nodes*	
None	181 (44.2)
1–3	109 (26.6)
4–9	70 (17.1)
≥10	49 (11.9)
*Posttherapy stage*	
0	61 (14.9)
I	92 (22.5)
II	129 (31.5)
III	127 (31.9)
*Adjuvant treatment*	
Nil	31 (7.6)
Hormonal therapy	125 (30.6)
Chemotherapy	54 (13.2)
Chemotherapy followed by hormonal therapy	105 (25.6)
Trastuzumab	94 (23.0)
*Radiotherapy*	
Yes	310 (75.8)
No	99 (24.2)
*Mastectomy*	
With radiation	101 (60.1)
Without radiation	67 (39.8)

### Survival

Median follow-up was 42.1 months (range 0.8–147.3 months). During follow-up, 25 (6.1 %) patients had local relapse, 84 (20.5 %) had distant metastases and 53 (13.0 %) died. We evaluated patients’ outcome in relation to different variables such as pCR, stage at surgery, tumor molecular subtype, use of Trastuzumab for HER2-positive tumors, type of surgery and breast radiotherapy. The occurrence of local relapse was not correlated with stage of disease at surgery, type of surgery and radiation therapy, while it was more frequent among patients with HR-negative tumors (*p* = 0.007 by Pearson’s *χ* ^*2*^).

**Fig. 1:**
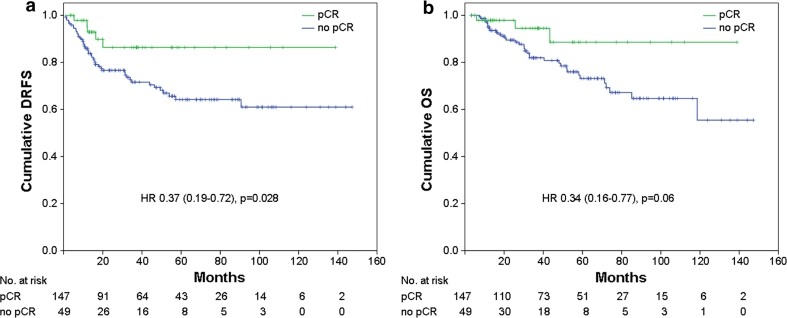
**a** Distant relapse free survival (*DRFS*) and **b** overall survival (*OS*) stratified by pathological complete response (*pCR*) for the whole population, excluding patients with HR-positive/HER2-negative tumors

**Fig. 2:**
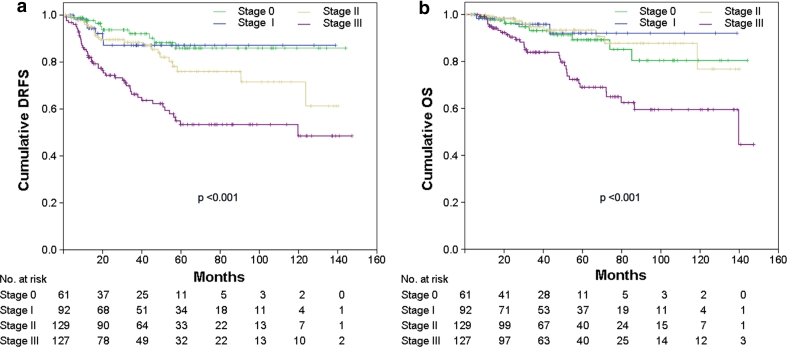
**a** Distant relapse free survival (*DRFS*) and **b** overall survival (*OS*) stratified by pathological stage after systemic neoadjuvant chemotherapy

At Kaplan–Meier analysis of the whole population, pCR was not found to be a prognostic factor for DRFS and OS (not shown). However, excluding from the analyses patients with luminal A or luminal B/HER2-negative tumors (a group of patients with favorable outcome, representing 51.9 % of the entire study population), pCR resulted predictive of better DRFS (*p* = 0.028: HR = 0.37, 95 % CI = 0.19–0.72) with a trend toward significance for OS (*p* = 0.06; HR = 0.34, 95 % CI = 0.16–0.77) (Fig. 1a, b). Patients with higher stage of disease after NST were more likely to develop distant metastasis and die from breast cancer (Fig. 2a, b; *p* < 0.001). Patients who achieved pCR (stage 0) had DRFS and OS rates of 87.1 % (95 % CI: 77.3–96.9 %) and 92.0 % (95 % CI: 84.8–99.2 %), respectively, similar to those of patients with surgical stage I (DRFS: 85.9 %; 95 % CI: 77.1–94.7 % and OS: 80.4 %; 95 % CI: 66.9–93.9 %). Stage II patients had DRFS rates of 61.3 % (95 % CI: 39.7–82.9 %) and OS rates of 76.8 % (95 % CI: 55.2–98.4 %), while stage III patients had DRFS rates of 48.5 % (95 % CI: 35.4–61.6 %) and OS rates of 44.6 % (95 % CI: 17.7–71.5 %).

**Fig. 3:**
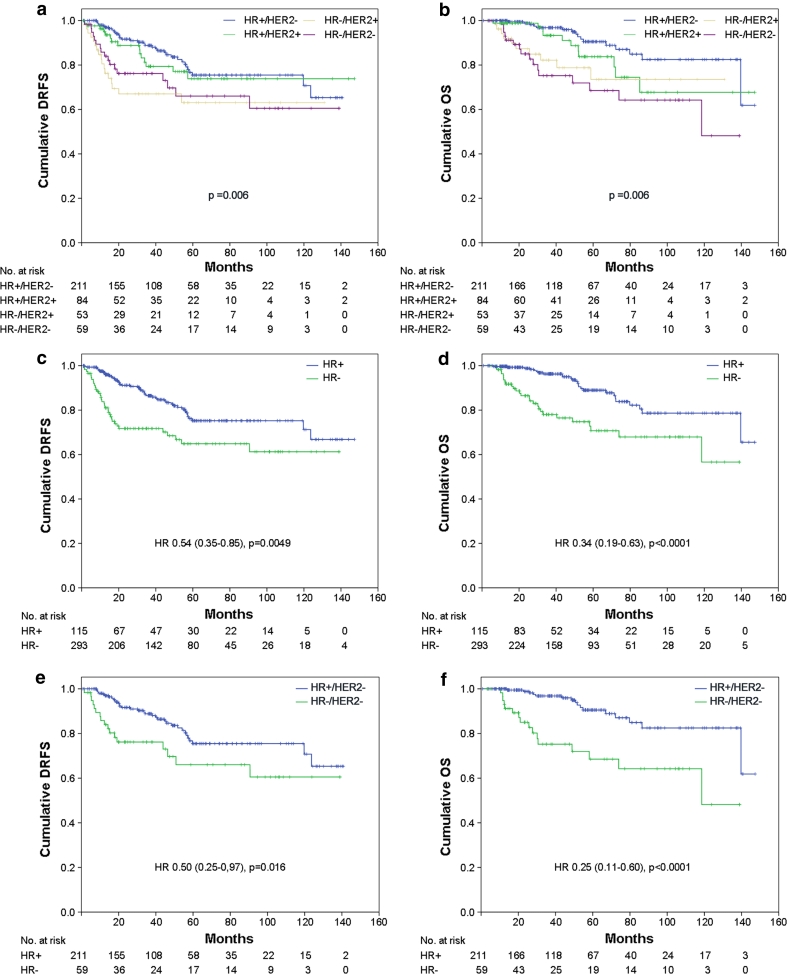
**a**, **c**, **e** Distant relapse free survival (*DRFS*) and **b**, **d**, **f** overall survival (*OS*) stratified by molecular subtypes for the whole population (**a**, **b**), by hormone receptor (*HR*) status (HR+ and HR−) for the whole population (**c**, **d**) and by HR status for patients with HER2-negative tumors (**e**, **f**)

Significant differences in DRFS (*p* = 0.006) and OS (*p* = 0.006) were observed among patients with different tumor molecular subtypes (Fig. 3a, b), the group of patients with luminal A or luminal B/HER2-negative tumors showing a better prognosis. Moreover, patients with HR-positive tumors had a significant longer DRFS (*p* < 0.005: HR = 0.54, 95 % CI = 0.35–0.85) and OS (*p* < 0.0001: HR = 0.34, 95 % CI = 0.19–0.63) compared with patients with HR-negative tumors (Fig. 3c, d). When survival analysis was stratified according to HER2 status, the DRFS and OS advantage for HR-positive tumors was limited to the HER2-negative population (*p* = 0.016: HR = 0.50, 95 % CI = 0.25–0.97 and *p* < 0.0001: HR = 0.25, 95 % CI = 0.11–0.60, respectively) (Fig. 3e, f), while among HER2-positive group, HR positivity was predictive of a longer DRFS (*p* = 0.044: HR = 0.50, 95 % CI = 0.24–1.0), but not OS (not shown). In our population, neither HER2 status nor the use of Trastuzumab in the HER2-positive patients was statistically associated with clinical outcome (not shown).

**Fig. 4:**
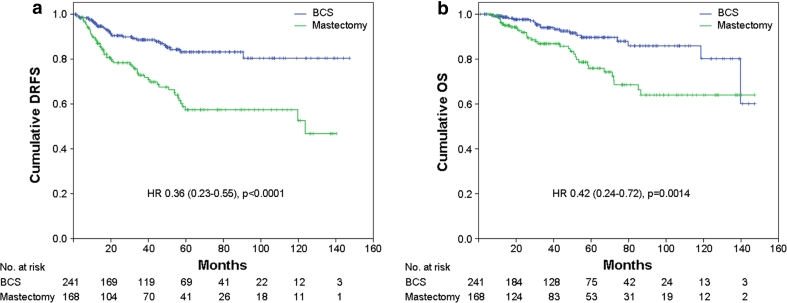
**a** Distant relapse free survival (*DRFS*) and **b** overall survival (*OS*) stratified by type of surgery. *BCS* breast conservative surgery

Patients who underwent BCS were more likely to have a better DRFS (*p* < 0.0001: HR = 0.36, 95 % CI = 0.23–0.55) and OS (*p* = 0.0014: HR = 0.42, 95 % CI = 0.24–0.72) compared with those who required mastectomy (Fig. 4a, b). No differences in survival were observed in patients treated with or without radiotherapy after surgery (not shown).

In multivariate analysis, the variables independently associated with shorter DRFS and OS were absence of HR expression (DRFS and OS: *p* < 0.001), mastectomy (DRFS: *p* = 0.009; OS: *p* = 0.05) and stage III disease (DRFS: *p* < 0.001; OS: *p* = 0.003), whereas the molecular subtype luminal B/HER2-positive tumors reached statistical significance for OS (*p* = 0.035), but not for DRFS (Tables 4, 5).

**Table 4:** Multivariable proportional hazard regression model predicting DRFS

	Parameter estimate	Hazard ratio	95 % CI	*p* value
*Age*				
>35 years			Reference	
≤35 years	0.158	1.171	0.457–3.003	n.s.
*Histologic type*				
Lobular			Reference	n.s.
Ductal	0.065	1.067	0.568–2.006	n.s.
Others	0.187	1.206	0.247–5.874	
*Molecular subtype preNST*				
Luminal A and B/HER2 negative			Reference	
Luminal B/HER2 positive	0.591	1.807	0.970–3.365	0.062
HER2 enriched	1.678	5.354	2.747–10.435	0.000
Triple negative	1.141	3.130	1.703–5.752	0.000
*Type of surgery*				
BCS			Reference	
Mastectomy	0.643	1.903	1.170–3.093	0.009
*Stage*				
0			Reference	
I	0.185	1.203	0.419–3.454	n.s.
II	0.751	2.119	0.825–5.443	n.s.
III	1.810	6.108	2.376–15.707	0.000

**Table 5:** Multivariable proportional hazard regression model predicting OS

	Parameter estimate	Hazard ratio	95 % CI	*p* value
*Age*				
>35 years			Reference	n.s.
≤35 years	0.178	1.195	0.404–3.533	
*Histologic type*				
Lobular			Reference	
Ductal	0.424	1.528	0.590–3.955	n.s.
Others	0.509	1.664	0.170–16.266	n.s.
*Molecular subtype preNST*				
Luminal A and B/HER2 negative			Reference	
Luminal B/HER2 positive	0.846	2.330	1.062–5.111	0.035
HER2 enriched	1.751	5.761	2.438–13.610	0.000
Triple negative	1.696	5.453	2.620–11.350	0.000
*Type of surgery*				
BCS			Reference	
Mastectomy	0.606	1.834	1.170–3.093	0.056
*Stage*				
0			Reference	
I	0.726	2.066	0.547–7.808	n.s.
II	0.466	1.593	0.409–6.205	n.s.
III	1.900	6.683	1.877–23.801	0.003

## Discussion

In this retrospective study, we show that HR negativity, requirement for mastectomy and pathological stage III disease are independently associated with a worse outcome in breast cancer patients treated with NST in clinical practice. These data are of high interest since they derive from a very heterogeneous group of patients, treated with different neoadjuvant/adjuvant regimens outside of clinical trials and with a long follow-up period.

During the course of the last 12 years, the adjuvant treatment of patients affected by early breast cancer is profoundly changed, going from first generation regimens like CMF and epirubicin–CMF, second generation regimens like FEC to third generation regimens, like EC followed by docetaxel (Sachelarie et al. 2006; Peto et al. 2012). Also, we delivered Trastuzumab in the adjuvant treatment of HER2-positive tumors from 2005 and in the neoadjuvant setting from 2006 (Romond et al. 2005; Arteaga et al. 2012). Finally, surrogate definitions of intrinsic subtypes with immunohistochemistry have only recently proven to be effective in defining prognosis and selecting adjuvant therapy in early stage breast cancer patients (Cheang et al. 2009; Nielsen et al. 2010; Goldhirsch et al. 2011).

In our study, high tumor grade, high proliferative activity, HR-negativity expression in tumor biopsy, the use of neoadjuvant Trastuzumab and an increase in number of chemotherapy cycles resulted significantly associated with higher rates of pCR at univariate analysis, consistent with current literature (Colleoni et al. 2009; Huober et al. 2010; Kim et al. 2010; Precht et al. 2010; Straver et al. 2010; Untch et al. 2011; von Minckwitz et al. 2011), but only HR negativity and neoadjuvant Trastuzumab were confirmed at multivariate analysis. The association between HR negativity and pCR has been observed also in a recently published meta-analysis based on 20 studies providing data with classification of HER2 positivity according to HR status (Houssami et al. 2012). Their estimates of pCR were 8.3 % in the luminal A and luminal B/HER2-negative subtype; 18.7 % in the luminal B/HER2-positive subtype; 38.9 % in the HER2-enriched subtype and 31.1 % in the triple negative subtype (Houssami et al. 2012).

However, although most neoadjuvant chemotherapy trials have shown that pCR is associated with a favorable outcome in terms of DRFS and OS (Kuerer et al. 1999; Kaufmann et al. 2006; Buzdar et al. 2007; Dawood et al. 2008), in our cohort pCR was not predictive of better prognosis. This discordance is not explained by the definition of pCR we applied, since it is now the most commonly used. Different definitions for pCR have been used in different clinical trials, varying according to site (i.e., breast only or both breast and axillary) and residual disease (i.e., presence of focal invasive cancer, non-invasive cancer residuals or absence of invasive and non-invasive cancer) (Sataloff et al. 1995; Bear et al. 2003; Green et al. 2005; von Minckwitz et al. 2010). Absence of invasive and non-invasive cancer has been reported to be associated with a better prognosis (von Minckwitz et al. 2010). The incidence of residual non-invasive cancer in our study (only six patients had residual in situ ductal carcinoma in the final pathologic examination), is too low to justify the poorer outcome observed in the whole population. More importantly, most of the patients in the study (211 patients, 51.9 %) had luminal A or luminal B/HER2-negative tumors, a subgroup considered to have slowly proliferating and less chemotherapy responsive tumors. In these patients, pCR has been shown to be not associated with prognosis (von Minckwitz et al. 2010). The high number of patients included in the luminal A or luminal B/HER2-negative subgroup in our study could have diluted the effect of pCR on outcome. Indeed, when these patients were excluded from the analyses, pCR was significantly associated with longer DRFS and OS.

The outcome of patients included in this study was significantly affected by stage at surgery, HR expression and type of surgery. Several studies have showed that a higher stage after NST is predictive of poor prognosis (Fisher et al. 1998; Cance et al. 2002; Carey et al. 2005). Consistently, we found that patients with stage III disease had a significantly shorter DRFS and OS. Lack of HR expression is another well-established parameter associated with poor prognosis (Osborne and McGuire 1979; McGuire et al. 1986). In our study, after a follow-up of about 12 years, patients with HR-negative tumors had a significantly lower rate of DRFS (61 vs. 67 %, *p* < 0.001) and OS (56.6 vs. 65.5 %, *p* < 0.001) compared with patients with HR-positive tumors, independently from HER2 status, at least for DRFS. The OS advantage for HR positivity was lost in the subgroup of HER2-positive tumors. This might be explained by the lower responsiveness of HR- and HER2-positive tumors to the effect of adjuvant endocrine therapy (De Placido et al. 2003; Massarweh et al. 2006).

BCS was carried out in 241 (58.9 %) patients and this is in agreement with the percentage of BCS performed after NST reported in clinical trials (Bear et al. 2006; Fisher et al. 1998; Alm El-Din and Taghian 2009). These patients had a significantly better prognosis in terms of DRFS and OS compared with patients who underwent mastectomy. Similar data are presented by other authors who related these findings to patients’ selection: patients were more likely to have BCS if they presented with earlier stage disease or a clinical complete or greater than 50 % partial response (Schwartz et al. 1994; Kuerer et al. 1999). In our cohort, this advantage was independent of age, histologic type, molecular subtype and surgical stage, but we agree that the achievement of BCS can be considered as an indirect measure of clinical response of the primary tumor, parameter not included in our multivariate analyses since we could not uniformly assess it throughout our patient population. In this study, clinical response was evaluated before, during and after neoadjuvant chemotherapy either by physical examination or by echographic and mammographic measurements, but the lack of standardization did not allow us to include clinical response as a variable for the multivariate analyses. Considering achievement of BCS as a surrogate marker of primary tumor response, our results suggest that the clinical response to NST is a strong predictive factor of good outcome. Some neoadjuvant trials have provided evidence of the prognostic value of clinical response, even when it was not correlated with pCR (Hortobagyi et al. 1988; Jacquillat et al. 1991; Cameron et al. 1997; Pierga et al. 2003).

During follow-up, 25 (6.1 %) patients had LRR, which was related neither to the type of surgery nor to radiotherapy. These data are in agreement with those of other authors reporting a rate of LRR ranging from 6 to 10 %, with a trend toward higher rates in patients with basal-like subtypes (Chen et al. 2004; Tanioka et al. 2010; Meyers et al. 2011; Min et al. 2011).

In conclusion, this retrospective neoadjuvant study, based on a population of patients treated in the practice of clinical medicine, shows that HR negativity, stage III disease at surgery and failure to achieve BCS after NST are independent factors negatively associated with prognosis. Moreover, the results of this study further confirm that pCR is of no prognostic value in patients with luminal A or luminal B/HER2-negative tumors. These patients, therefore, should not be included in neoadjuvant clinical trials whose primary end point is pCR, as suggested by other authors (Eiermann et al. 2001; Berruti et al. 2011).
